# miR-548x and miR-4698 controlled cell proliferation by affecting the PI3K/AKT signaling pathway in Glioblastoma cell lines

**DOI:** 10.1038/s41598-020-57588-5

**Published:** 2020-01-31

**Authors:** Mohammad Reza kalhori, Ehsan Arefian, Fereshteh Fallah Atanaki, Kaveh Kavousi, Masoud Soleimani

**Affiliations:** 1 Medical Biology Research Center, Health Technology Institute, Kermanshah University of Medical Sciences, Kermanshah, Iran; 2 Molecular Virology lab, Department of Microbiology, School of Biology, College of Science, University of Tehran, Tehran, Iran; 3 Laboratory of Complex Biological Systems and Bioinformatics (CBB), Department of Bioinformatics, Institute of Biochemistry and Biophysics (IBB), University of Tehran, Tehran, Iran; 4 Department of Hematology, Faculty of Medical Sciences, Tarbiat Modares University, Tehran, Iran

**Keywords:** Cancer therapy, miRNAs, RNAi

## Abstract

Glioblastoma multiforme (GBM) is the most aggressive and prevalent form of brain tumor cancers that originate from glial cells. This study proposed to investigate the effect of miR-548x and miR-4698 on the proliferation and the PI3K/AKT signaling pathway in glioblastoma cell lines. The molecular features of glioblastoma were studied using KEGG and TCGA sites. Next, by using miRwalk 2.0 and TargetScan version 7.1, the microRNAs that target critical genes in the PI3k/AKT pathway were selected according to score. The pre-miR-548x and pre-miR-4698 were cloned in a pCDH plasmid to produced lentiviral vector. The expression levels of miR-548x, miR-4698 and target genes were detected by qRT-PCR. The MTT, cell cycle, annexin and colony formation assay was used to detect the cell proliferation. MiR-548x and miR-4698 predicted target genes (Rheb, AKT1, mTOR, PDK1) were also evaluated by luciferase assay. The expression of AKT was detected by western blotting. Our results described that overexpression of miR-548x and miR-4698 could inhibit proliferation of A-172 and U251 cells. Also, miR-548x promoted the cell cycle arrest of GBM cell lines. The luciferase reporter assay results showed the 3′ UTR of PDK1, RHEB, and mTOR are direct targets of the miR-548x and miR-4698. Too, the western blot analysis revealed that miR-548x and miR-4698 could downregulate the AKT1 protein expression. Overall, our findings suggest that miR-548x and miR-4698 could function as tumor suppressor genes in glioblastoma by controlling the PI3K/AKT signaling pathway and may act as gene therapy for clinical treatment of glioblastoma multiforme.

## Introduction

Cancer is a genetic disease and mainly caused by activation of oncogenes or disruption of tumor suppressor genes^1^. In recent years, various treatment methods have been used for cancer. Some of the most common include surgery, radiotherapy, chemotherapy, hormone therapy and immunotherapy^2^. Glioblastoma multiforme (GBM) is the most aggressive and prevalent form of brain tumor cancers that originate from glial cells. Despite current treatment for GBM including surgical resection followed by the chemo-radiotherapy unfortunately the median survival rate of the patient is approximately 16.9 months. Therefore, the new therapeutic strategy needs to be identified for GBM therapy^3,4^. One of the common molecular events of glioblastoma is PTEN mutation, which leads to increased activity of the PI3K/AKT pathway. So that glioblastoma cells exhibit a high level of AKT activity^5^.

Gene therapy is the transfer of genes to cells using viral or non-viral vectors. Gene therapy will play an essential role in the treatment of cancer in combination with other therapies such as surgery, chemotherapy and radiation therapy^6^. One of the molecular methods used in treating cancer is the interfering RNAs such as microRNA and siRNA. These molecules can, by binding to the 3′UTR of their target genes, reduce the function of the genes^7,8^. In the miRBase database, approximately 68 members of the miR-548 family have been reported that are discovered in all the human chromosomes. The miR-548 family has a transposon element origin and plays an essential role in various biological processes^9,10^. Hermansen *et al*. showed that some miRNAs are capable of determining the prognosis of patients with glioblastoma. They used miRNome screening on 40 samples from glioblastoma patients. They found four miRNAs: miR-548x, miR-331-3p, miR-3125, and miR-107 were capable predicted short- and long-term survival up 78%, and all of these miRNAs were downregulated in GBM. Their investigation showed that low expression of these microRNAs were significantly correlated with short term survival^11^. He *et al*.; in 2017 used next-generation sequencing (NGS) and qRT-PCR to analyze the miRNA expression profile of adenocarcinomas tumor compared adjacent non-tumor tissue. Their result showed that twenty-three miRNAs have differential expression. More investigations showed miR-548x, and other 17 miRNAs downregulated in adenocarcinoma samples compared to adjacent non-tumor tissue^12^. In the study published by Waseem *et al*., they investigated prostate cancer tissue using a microarray. Their results showed that the expression of miR-711, miR-1246 and miR-548x-3 decreased in cancerous tissue^13^.

The miR-4698 is located on chromosome 12 and associated with Conjunctival Cancer^14^. Liu *et al*., in 2014, investigated the expression profile of miRNAs by microarrays in gastric CSCs. The result showed that miR-4698 and 172 other microRNAs were under-expressed in gastric CSCs, indicating these miRNAs may have tumor suppressors function^15^. Also, Shen *et al*. investigated the altered microRNA expression and the involvement of miRNAs in the pathophysiology of chronic obstructive pulmonary disease (COPD). They found the hsa-miR-4698, and several miRNAs significantly downregulated in each lung tissues from COPD cases compared to healthy tissues^16^.

According to our bioinformatics studies in the TargetScan 7.1 and miRwalk 2.0 database, we predicted that some miRNAs, as well as miR-548x and miR-4698 could target some genes that are overactive in the PI3K/AKT pathway (PI3KCB, PI3KCA, PDK1, AKT1, Rheb, MDM2, mTOR, and CREB1) in glioblastoma. Therefore, it is expected that these microRNAs by inhibiting cell proliferation, induction of the cell cycle arrest and apoptosis can be utilized as the molecular therapeutics for GBM. This study proposed to investigate the influence of miR-548x and miR-4698 overexpression on PI3K/AKT signaling pathway and cellular behavior as gene therapy in glioblastoma cell lines. We studied the biological functions by MTT assay, Annexin V, colony formation assay and cell cycle assay. Furthermore, molecular evolution and genes expression change were studied by qRT-PCR, western blotting analysis and Luciferase Reporter Assay. In this study we investigate the effect of miR-548x and miR-4698 for the gene therapy in the glioblastoma cell line for the first time.

## Material and Method

### Bioinformatics study

The molecular features of glioblastoma were studied using KEGG and TCGA sites. The critical effector genes in the AKT pathway, which regulate cell proliferation, were identified and selected. First, to determine whether these genes are overexpressed in glioblastoma or not, we examined their expression using TCGA. For this purpose, the RPKM values for normal and cancer samples were downloaded from the TCGA database through R package “TCGAbiolinks”, and the expression of the target genes (Rheb, AKT1, MTOR, CREB1, MDM2, and PDK1) were extracted. This data includes 5 normal and 169 tumor GBM samples. The significance of variations in the expression of these genes in the healthy and GBM groups was assessed by unpaired t-test. Next, by using miRwalk 2.0 and TargetScan 7.1, the microRNAs that target these critical genes were selected according to Context++ score percentile (Tables 1, 2). Then the expression changes of miR-548x, miR-4698, were investigated by GEO and TCGA in cancer tissue compared to healthy tissue. Finely genetic features of each of the glioblastoma cell lines (A-172 and U251) were analyzed using the ATCC site.

**Table 1:** List of the miR-548x predicted target gene by the TargetScan 7.1 database.

	The predicted consequential pairing of the target region (top) and miRNA (bottom)	Site type	Context++ score	Context++ score percentile
Position 5089–5095 of PIK3CA 3′ UTR hsa-miR-548x-3p	5’...UUUUUAUUUAAUUUU-GUUUUUAU... ||||| |||||| 3′ CUUUCAUUAACGUCAAAAAU	7mer-A1	−0.07	95
Position 2238–2245 of PIK3CA 3′ UTR hsa-miR-548x-5p	5’...ACAAAAUUUAUACCACUUUUGCA... ||||||| 3’ GUUUUUGACGUUAAUGAAAACGU	8mer	−0.04	70
Position 816–823 of PIK3CB 3′ UTR hsa-miR-548x-3p	5’...GGCACAUAUUAAUAC-AGUUUUUA... |||| ||||||| 3’ CUUUCAUUAACGUCAAAAAU	8mer	−0.03	84
Position 76–82 of AKT1 3′ UTR hsa-miR-548x-3p	5’...GAUCUGUAUUUAAUGGUUUUUAU... ||| |||||| 3’ CUUUCAUUAACGU-CAAAAAU	7mer-A1	−0.03	49
Position 3443–3449 of AKT1 3′ UTR hsa-miR-548x-5p	5’...UCAUUAAAAGUCUCACUUUUGCU... ||||||| 3’ GUUUUUGACGUUAAUGAAAACGU	7mer-m8	−0.02	52
Position 3658–3664 of PDK1 3′ UTR hsa-miR-548x-5p	5’...ACUGUGUGAAGAAUUUUUUGCAU... |||||| 3’ GUUUUUGACGUUAAUGAAAACGU	7mer-A1	−0.07	83
Position 3849–3855 of PDK1 3′ UTR hsa-miR-548x-3p	5’...AAAGACAAGAUUGUCAGUUUUUG... ||||||| 3’ CUUUCAUUAACGUCAAAAAU	7mer-m8	−0.02	67
Position 1018–1025 of RHEB 3′ UTR hsa-miR-548x-5p	5’...UUUUUAAACGAUUCCCUUUUGCA... ||||||| 3’ GUUUUUGACGUUAAUGAAAACGU	8mer	−0.22	97
Position 152–159 of CREB1 3′ UTR hsa-miR-548x-5p	5’...UUUCAUUCAUUUGUGCUUUUGCA... ||||||| 3’ GUUUUUGACGUUAAUGAAAACGU	8mer	−0.07	82
Position 4313–4319 of CREB1 3′ UTR hsa-miR-548x-3p	5’...GACUUUUUAAAUGGAAGUUUUUC... ||| ||||||| 3’ CUUUCAUUAACG–UCAAAAAU	7mer-m8	−0.02	67
Position 688–694 of PTK2 3′ UTR hsa-miR-548x-3p	5’...ACAGAAUAAUGUGCCAGUUUUUU... |||| ||||||| 3’ CUUUCAUUAACG––UCAAAAAU	7mer-m8	−0.02	67

**Table 2:** List of the miR-4698 predicted target gene by the TargetScan 7.1 database.

	The predicted consequential pairing of the target region (top) and miRNA (bottom)	Site type	Context++ score	Context++ score percentile
Position 1482–1489 of PIK3CA 3′ UTR hsa-miR-4698	5’...GGACACAUUCUUAAACAUUUUGA... ||| ||||||| 3’ ACCCCAGAAGGAGAUGUAAAACU	8mer	−0.03	75
osition 47–53 of PIK3CB 3′ UTR hsa-miR-4698	5’...GUUUCAUUUCAUUUUCAUUUUGC... ||| ||||||| 3’ ACCCCAGAAGGAGAUGUAAAACU	7mer-m8	−0.05	86
Position 106–112 of MTOR 3′ UTR hsa-miR-4698	5’...UUGACUUUGUUAAAUAUUUUGAA... ||||||| 3’ ACCCCAGAAGGAGAUGUAAAACU	7mer-A1	−0.03	71
Position 652–658 of AKT1 3′ UTR hsa-miR-4698	5’...UAUGUUGUUCAAAUGCAUUUUGG... ||| ||||||| 3’ ACCCCAGAAGGAGAUGUAAAACU	7mer-m8	−0.02	59
Position 6010–6016 of PDK1 3′ UTR hsa-miR-4698	5’...GAAGAUCCAUCUAAGAUUUUGAC... ||| |||||| 3’ACCCCAGAAGGAGAUG--UAAAACU	7mer-A1	−0.11	95
Position 3939–3945 of CREB1 3′ UTR hsa-miR-4698	5’...AAUUGGUCUCUGUUU--AUUUUGAA... ||||| |||||| 3’ ACCCCAGAAGGAGAUGUAAAACU	7mer-A1	−0.13	96
Position 951–957 of PTK2 3′ UTR hsa-miR-4698	5’...CCAUCCAUUCUGUUA-AUUUUGAA... ||| |||||| 3’ ACCCCAGAAGGAGAUGUAAAACU	7mer-A1	−0.07	90

### Cell culture

The cell lines human embryonic kidney (HEK293T), A-172 and U251 were purchased from Iranian Biological Resource Center (IBRC). All cell lines were grown in Dulbecco’s Modified Eagle’s Medium (DMEM) (Gibco, Grand Island, USA) and 10% fetal bovine serum (FBS) and antibiotics (100U/ml penicillin and 100 ng/ml streptomycin) in a humidified atmosphere containing 5% CO_2_ at 37 °C.

### Lentivirus packaging and cell transduction

The pre-miR-548x and pre-miR-4698 were cloned using restriction enzymes EcoRI/BamHI and NheI/BamHI respectively (Thermo Fisher Scientific, Waltham, USA) in the plasmid pCDH (System Biosciences, Palo Alto, USA). The recombinant plasmids or scramble with the pMDG plasmid (containing vsv-g) and the psPAX2 plasmid (packaging plasmid) were transfected to Hek293T cells by utilizing Lipofectamine 2000 (Invitrogen, Carlsbad, CA). By ultracentrifuge lentiviral supernatants were concentrated at 40000 g for 2 h. Then the glioblastoma cell lines were transduced by the concentrated viral particle (MOI = 5 and 4 µg/ml polybern). Forty-eight hours later, positive GFP cells were examined by flow cytometry (BD Biosciences, Franklin Lakes, USA) and fluorescence microscope.

### Gene expression analysis

After 72 h of transduction total RNA was isolated from U251 and A-172 cell lines using the TRIzol reagent (Invitrogen, Carlsbad, CA) according to the company instructions. Complementary DNAs (cDNAs) were manufactured with M-MuLV Reverse Transcription enzyme (Thermo Fisher Scientific, USA) with stem-loop RT primers (for miRNAs and SNORD47) and random hexamers (for genes)^17^. The Real Time-PCR reactions were done using the RealQ Plus 2x Master Mix Green (Ampliqon, Odense M, Denmark) according to the company instructions. Also, we used the 2-ΔΔCt method to measure the expression levels of miRNAs and target genes with B2M (beta-2-microglobulin) and SNORD47 as the endogenous controls. Primer sequences are listed in Table 3.

**Table 3:** List of primer sequences employed in this research.

Beta-actin*	F: CTT CTT TCC TGG GCA TG R: GTC TTT GCG GAT GTC CAC
PTK2*	F: ATA GAA CTT GGA CGA TGT ATT GG R: TGA CGC ATT GTT AAG GCT TC
PIK3CB*	F: ACT TGG TAA TCG GAG GAT AGG R: GAG TGC TTC AAC CTG CTT AG
PDK1*	F: ATC ACC AGG ACA GCC AAT AC R: CCT CGG TCA CTC ATC TTC AC
CREB1*	F: GTG AAC GAA AGC AGT GAC G R: ATA GAT ACC TGG GCT AAT GTG G
mTOR*	F: ATT CCG ACC TTC TGC CTT C R: TTG CCT TCT GCC TCT TAT GG
AKT1*	F: TGG CAC CTT CAT TGG CTA C R: GTC TGG ATG GCG GTT GTC
Rheb*	F: GAC TCC TAC GAT CCA ACC ATA G R: CGG CTG TGT CTA CAA GTT G
CCND1*	F: CCC TCG GTG TCC TAC TTC AAA TG R: CCT CCT CGC ACT TCT GTT CC
CDK2*	F: CGG AGC TTG TTA TCG CAA ATG C R: TGG CTT GGT CAC ATC CTG GAA G
CDK6*	F: GCG ACT TGA AGA ACG GAG R: ATC AAA CAA CCT GAC CAC G
PIK3CA*	F: CTC CTC TAA ACC CTG CTC ATC R: CAT ATC TTG CCGTAA ATC ATC C
miR-548X†	F: CGG AAT TCC CTC AGC CAT ACT ACC TC R: CGG GAT CCA CAT GCC ATT AGT AAC AAC G
miR-4698†	F: CTA GCT AGC CTA ATG AAC TCA GCA CCA AGG R: CGG GAT CCG GAA CCC AAA GAC AAC TAA GG

^*^Primers used for qRT- PCR. †Primers used for cloning miR-548x and 4698 in pCDH plasmid. F: Forward primer. R: Reverse primer.

### Cell viability and proliferation assay

For the cell viability assay, 48 h after transduction of A-172 and U251 cells approximately 5000 (cells/well) of controls and transduced cells were seeded in 96-well plates. Then at 72, 96 and 120 h post-transduction the cells were treated with 10 µl MTT reagents (5 mg/ml) (MTT, Sigma, St. Louis, USA) per well and maintained for 3 hours at 37 °C. Then the culture media were discarded, and 100 µl of DMSO was added to each well; furthermore absorbance was measured at 570 nm with a microplate reader (BioTek, Winooski, USA). All tests were performed in triplicate. The proliferation assay was done 48 h after transduction of A-172 and U251 cells. Approximately 1.5 × 10^4^ (cells/well) were cultured in 24-well plates and cultured at 37 °C in a 5% CO_2_ incubator. Then at 72, 96, 120 and 168 h post-transduction, the cells were fixed for 30 min in methanol and stained with 0.1% crystal violet. Stained cells were rinsed twice with PBS and allowed to air-dry. Ethanol was then added to dissolve the crystal violet. Absorbance was measured at 570 nm with a microplate reader (BioTek, Winooski, USA). All tests were performed in triplicate.

### Colony formation assay

For assessing the effect of miRNA-548x and miR-4698 on ability of cells to generate colony the colony formation assay was done. Forty-eight hours after transduction of U251 and A-172 cells, approximately 1000 cells were cultured in each well of a 6-well plate and maintenancefor two weeks. For the assay, cells were fixed by methanol (Merck, Darmstadt, Germany) and then were dyed with 0.1% crystal violet. By using ImageJ software, the colony formation rate was determined^18^. All experiments were done in triplicate.

### Luciferase reporter assay

To this purpose the wild-type 3ʹUTRs of the AKT1, Rheb, PDK1, mTOR genes were cloned using restriction enzymes XhoI and NotI (Thermo Fisher Scientific, USA) in the psiCHECK dual luciferase reporter vector (Promega, USA). For luciferase reporter assays, about 8 × 10^3^ (cells/well) HEK293T cells were cultured in 96-well plate and the following day were co-transfected with psiCHECK-3ʹUTR and miR-4698, miR-548x or scramble by utilizing Lipofectamine 2000 (Invitrogen, CA). Forty-eight hours after co-transfection the cells were collected, and Luciferase activity (Firefly or Renilla) was evaluated by Dual-Luciferase Reporter Assay System kit (Promega, USA) according to the company protocol. The Renilla luciferase activity was normalized by the Firefly luciferase activity. All examinations were done in triplicate.

### Western blot

We evaluated the effects of miRNA-548x and miR-4698 on the expression of AKT1 protein level in GBM cell lines A-172 and U251. Seventy-tow hours after transduction, the protein was extracted from cells by using RIPA lysis buffer including the protease inhibitor cocktail (Merck, Darmstadt, Germany) and concentration was evaluated using the BCA assay (Thermo Fisher Scientific, Waltham, USA). Next, equal concentrations of each specimen were separated by 10% sodium dodecyl sulfate-polyacrylamide gel electrophoresis and transferred to the polyvinylidene difluoride (PVDF) membrane. Following blocking with 5% skim milk (Merck, Darmstadt, Germany), the membrane was stored at 4 °C for overnight in the presence of primary antibodies anti-AKT1 sc-5298 (1:200, Santa Cruz Biotechnology Inc., Dallas, USA) or anti-B-actin (1:1000, Abcam, Cambridge, Britain) as the internal reference. Subsequently, the membrane was incubated with secondary antibodies conjugated with horseradish peroxidase (1:1000, Abcam, Cambridge, Britain). Lastly, for imaging, the ECL Western Blotting Substrate (Thermo Fisher Scientific, Waltham, USA) was used. The relative density of the band was analysis by Image J software version 1.52a^17^.

### Apoptosis study by Annexin-PE/7AAD

For assessing the effect of miRNA-548x and miR-4698 on the induction of apoptosis in transduced cells, at 96 h of post-transduction the annexin-V kit (BD Biosciences, USA) was utilized according to the manufacturer’s instruction. Briefly, the cells first were washed with binding buffer. Next, cells were dyed with 5 μl of AnnexinV PE and then with 5 μl of 7AAD. Finally, the apoptosis of the cells was estimated by flow cytometry (BD Biosciences, USA)

### Cell cycle analysis

For assessing the effect of miRNA-548x and miR-4698 on cell progression, at 72 and 96 h post-transduction, the cells (2-3 × 10^5^ cells) were rinsed with PBS and fixed using cold ethanol and stored at −20 °C overnight. Next, the cells were rinsed once with PBS and subsequently stained by 200 μl PI (50 μg/ml) and RNase (Thermo Fisher Scientific, USA). Subsequently, the cell cycle was analyzed by flow cytometry (BD Biosciences, USA).

### Statistical analysis

For statistical analysis, the GraphPad Prism 7.04 software (GraphPad Software, SanDiego, CA) was used, and all data were presented as mean ± standard deviation. One-way ANOVA determined differences between 3 groups (miRNA, scramble, and control) and the Student T-test determined differences between 2 groups (miRNA and scramble) in qRT-PCR. *P* < *0.05* was considered a statistically significant difference.

## Results

### Overexpression of miRNA-548x or miR-4698 inhibited cell viability, cell proliferation, and colony formation

**Figure 1:**
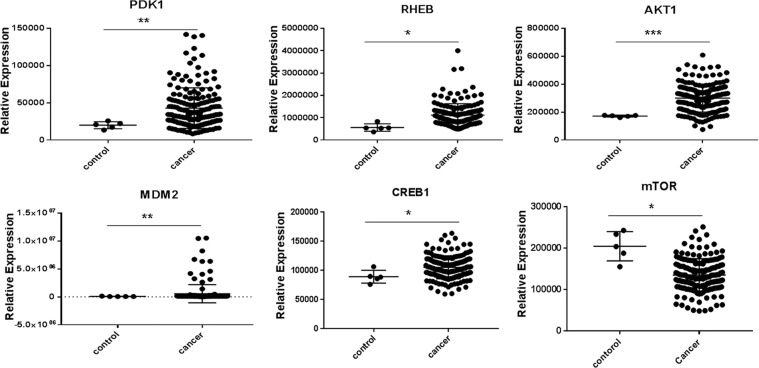
Investigation of the expression level of miR-548x and miR-4698 target genes in glioblastoma tumor tissue using TCGA. The relative expression changes of PDK1, RHEB, AKT1, MDM2, CREB1, mTOR showed that all of them expected mTOR were upregulated in GBM samples compared to healthy samples. *P < 0.05, **P < 0.01, ***P < *0.001* is considered as a significant level.

**Figure 2:**
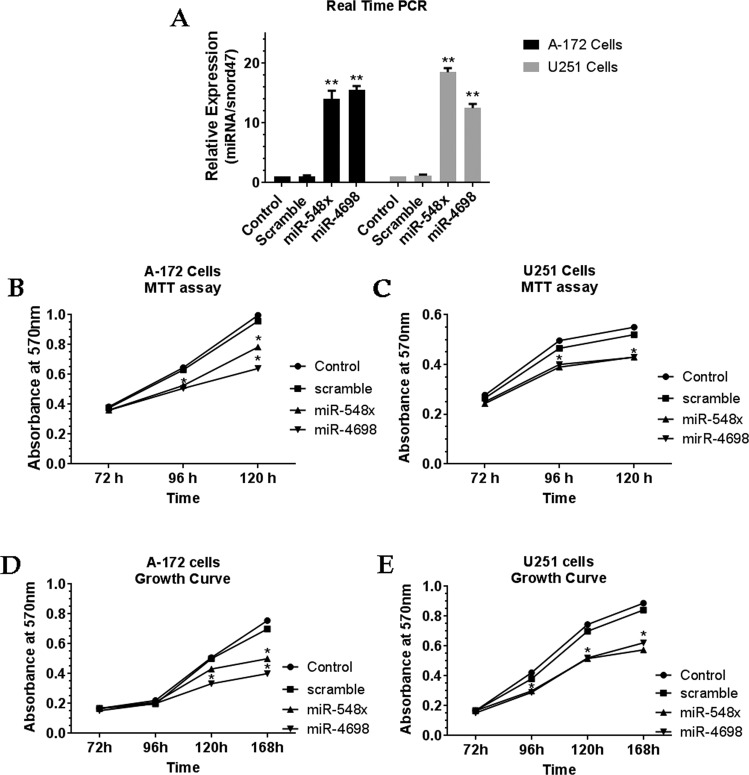
Overexpression of miR-548x and miR-4698 inhibited the cell proliferation of human glioblastoma cell lines. (**A**) QRT-PCR detected the relative expression of miR-548x and miR-4698 in U-251 and A-172 cells after 72 h of transduction (^∗∗^*P* < *0.01*). The miR-548x and miR-4698 were able to decrease the cell viability of A-172 (B) and U251 (**C**) cells at the point time 72, 96, 120 h post-transduction. Cell growth curves of A-172 (**D**) and U251 (**E**) cells transduced with miR-548x and miR-4698 decreased after 120, 168 h after transduction compared with the respective controls. Data are presented as mean ± SD of results from 3 independent investigations, (^∗^*P* < *0.05*) as compared with the control.

Our bioinformatics studies by Targetscan 7.1 and miRwalk 2.0 site have predicted that miR-548x and miR-4698 could simultaneously target 3ʹUTR of PI3KCB, PI3KCA, PDK1, AKT1, mTOR, MDM2, Rheb, and CREB1 genes that some of them were upregulated in glioblastoma sample according TCGA deta (Tables 1, 2). Also, the results of the TCGA showed that the expression of these miR-548x and miR-4698 target genes were significantly decreased in 169 tumor GBM samples compared to 5 healthy tissue (Fig. 1, P < 0.01). Therefore, down regulated of them could help to treatment of GBM. Moreover to investigated the biological function of miRNA-548x and miR-4698 in glioblastoma cell lines, we first up-regulated miRNA-548x or miR-4698 in A-172 and U251 cells by Lentivirus. The qRT-PCR analysis showed the ectopic expression of miR-548x and miR-4698 have significantly happened in A-172 and U251 cells as compared with control and scramble (Fig. 2A, *P* < *0.01*).

**Figure 3:**
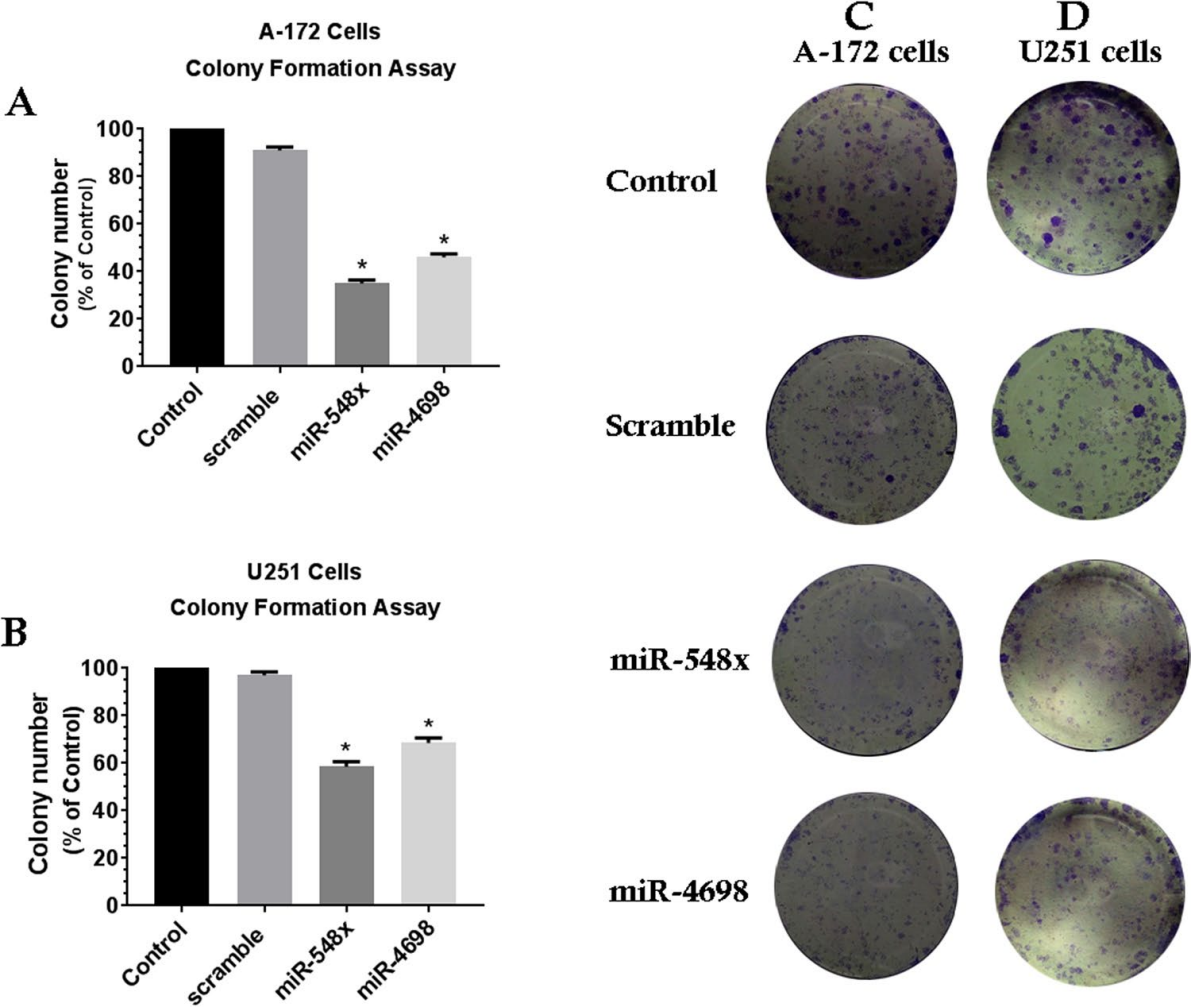
Overexpression of miR-548x and miR-4698 restrained the growth of human glioblastoma cell lines. Colony formation ability was decreased by overexpression of miR-548x or miR-4698 in A-172 (**A,B**) and U251 (**C,D**) cell lines compared with the respective controls. Data are presented as mean ± SD of results from 3 independent investigations, (^∗^*P* < *0.05*) as compared with the control.

Then MTT assay and proliferation assay showed that overexpression of miRNA-548x and miR-4698 could significantly decrease cell viability and prevent cellular proliferation in both A-172 and U251 cell lines compared with the control and scramble group (Fig. 2B–E, *P* < *0.05*). We next assessed whether the miRNA-548x and miR-4698 could regulate colony formation in GBM cell lines. The result of colony formation assay revealed that up-regulation of miRNA-548x and miR-4698 were able to repress cell growth and consequently reduce colony numbers of A-172 and U251 cell lines compared to the control and scramble (Fig. 3A–D, *P* < *0.05*). Overall, our results revealed that the miRNA-548x and miR-4698 have a suppressor effect on the biological functions of GBM cell lines.

### MiR-548x and miR-4698 directly target several genes of PI3K/AKT signaling pathway in human Glioblastoma cell lines

**Figure 4:**
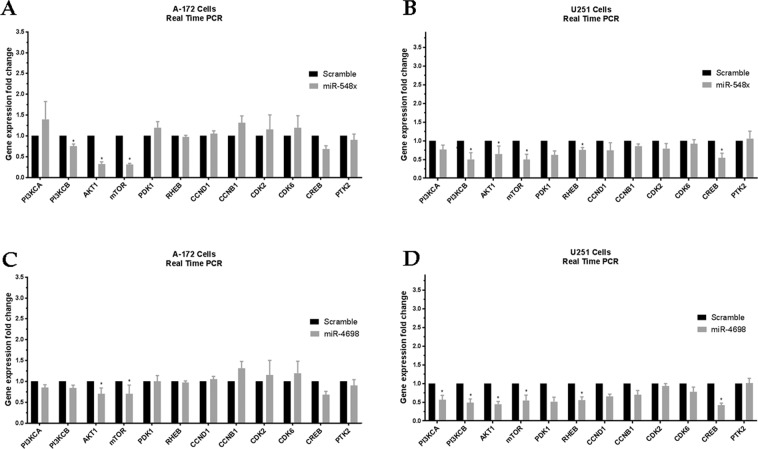
The PI3K/AKT pathway genes expressions were changed by overexpression of miR-548x or miR-4698. The qRT-PCR was used to investigate gene expression in A-172 (**A**) and U251 (**B**) cell line after 72 h of miR-548x or miR-4698 overexpression compared with the respective control. Data are presented as mean ± SD of results from 3 independent investigations, (^∗^*P* < *0.001*) as compared with the scramble. Results were normalized using B2M as an internal reference gene.

**Figure 5:**
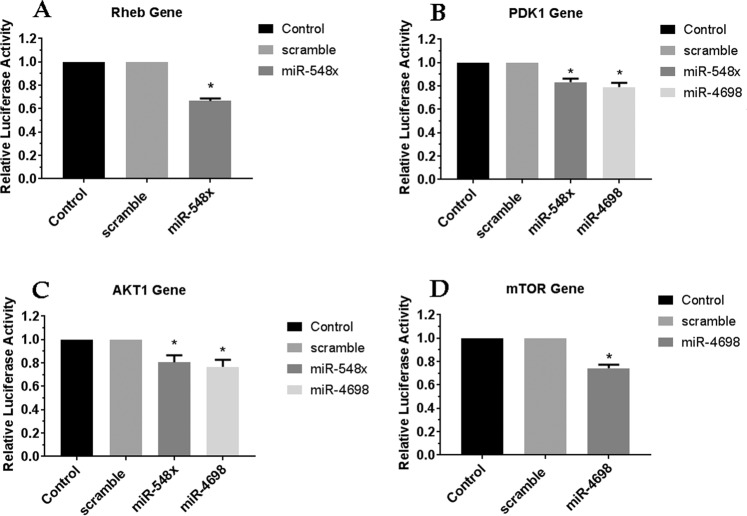
MiR-548x and miR-4698 targeted 3ʹUTR of mTOR, Rheb, AKT1, and PDK1 genes. The Dual Luciferase Reporter Assay was applied 48 h post-transfection to study the intercommunication between miR-548x or miR-4698 seed site with 3ʹUTR of target genes. MiR-548x ectopic expression could significantly inhibit the luciferase activity of Rheb (**A**), PDK1 (**B**) and AKT1 (**C**) genes and miR-4698 ectopic expression could significantly inhibit the luciferase activity of PDK1 (B), AKT1 (**C**) and mTOR (**D**) genes in HEK293T cell compared with the respective control. Data are presented as mean ± SD of results from 3 independent investigations, (^∗^*P* < *0.05*) as compared with the control.

To investigated the rule of miR-548x and miR-4698 on the expression of predicted target genes, we applied qRT-PCR. The results showed that the ectopic expression of miR-548x was able to significantly down-regulate the expression of AKT1 and mTOR in A-172 cells and also PI3KCB, AKT1, mTOR, Rheb and CREB genes in U251 cell lines (Fig. 4A,B, *P* < *0.05*). Moreover, the results revealed that the ectopic expression of miR-4698 was able to significantly down-regulate the expression of AKT1 and mTOR in A-172 cells and also PI3KCA, PI3KCB, AKT1, mTOR, Rheb and CREB genes in U251 cell lines (Fig. 4C,D, *P* < *0.05*). For additional research, we utilized dual Luciferase reporter assays to investigate the interaction of miR-548x and miR-4698 with 3ʹUTR of PDK1, AKT1, Rheb, and mTOR genes. We observed that miR-548x could significantly reduce the luciferase activity for 3ʹUTR of Rheb, PDK1 and AKT1 genes as compared with control and scramble (Fig. 5A–C, p < 0.05). Also, miR-4698 was able to reduce the luciferase activity for 3ʹUTR of PDK1, AKT1 and mTOR genes as compared with control and scramble (Fig. 5B–D, p < 0.05).

### MiR‐548x and miR-4698 directly targets AKT1 and could decrease their protein expression

**Figure 6:**
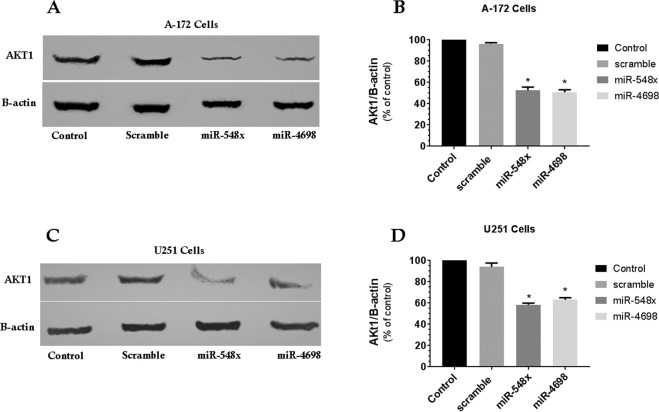
MiR-548x and miR-4698 inhibited AKT1 protein expression in human glioblastoma cell lines. Protein expression was investigated 72 h post-transduction by western-blot analysis. Overexpression of miR-548x or miR-4698 could decrease the protein expression of AKT1 in A-172 (**A**) and U251 (**B**) cell lines compared with the control cells. Also, western blot analysis indicated a reduction in protein level of AKT1 in A-172and U251 (**C,D**) cell lines compared with the respective control. Protein normalization was achieved with β-actin for AKT1. Data are presented as mean ± SD of results from 3 independent investigations, (^∗^*P* < *0.05*) as compared with the control.

The AKT1 gene goes out of control in many cancers and plays a central role in the PI3K pathway. Consequently, targeting it can play a significant role in the treatment of cancer (Bellacosa, Testa, *et al*. 2004). Additionally, our investigation in TCGA showed that the AKT gene was upregulated in GBM tissue compared to healthy tissue (Fig. 1), So we performed western blot analysis to understand the effect of miR-548x and miR-4698 on AKT1 protein expression in GBM cell lines. The event has shown that ectopic expression of miR-548x or miR-4698 significantly decreased the protein expression of AKT1 in U251 and A-172 cells (Fig. 6A–D, *P* < *0.05*). Therefore, we presume that miR‐548x and miR-4698 may help as a potential curative target in the treatment of GBM.

### Overexpression of miRNA-548x or miR-4698 repressed the cell cycle and apoptosis of GBM cell lines

**Figure 7:**
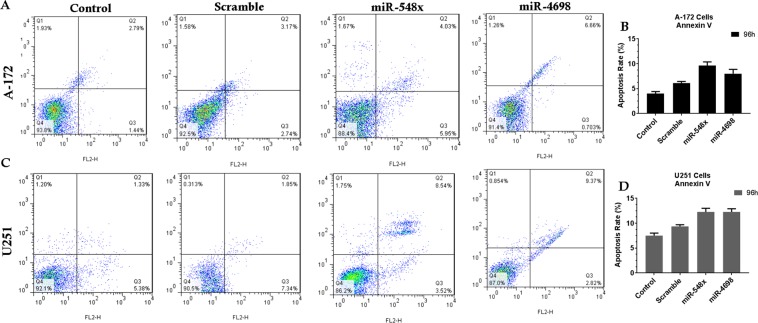
MiR-548x or miR-4698 and induction of the apoptosis in human glioblastoma cell lines. The flow cytometry result of Annexin V staining exhibited that miR-548x or miR-4698 could not significantly increase apoptosis in A-172 (**A,B**) and U251 (**C,D**) cell lines compared with the respective control 96 h post-transduction. Data are presented as mean ± SD of results from 3 independent investigations, (^∗^*P* < *0.05*) as compared with the control.

To confirm the tumor suppressor effects of miRNA-548x and miR-4698, the Annexin V staining and flow cytometry were performed in transducted A-172 and U251 cell lines. Results showed that ectopic expression of miRNA-548x was not able to promote apoptosis (early and late apoptotic cells) in the A-172 cells (9.6 ± 1.6% vs. 5.8 ± 1.5%) and U251 cells (12.1 ± 1.7% vs. 9.1 ± 1.6%) at 96 h post-transduction as compared to scramble group (Fig. 7A–D, *P* < *0.05*). Also, the same results were found about miR-4698 and this miRNA could not induce apoptosis in the A-172 cells (7.5 ± 1.3% vs. 5.8 ± 1.5%) and U251 (12.2 ± 1.2% vs. 9.1 ± 1.6%) cell lines (Fig. 7A–D, *P* < *0.05*).

**Figure 8:**
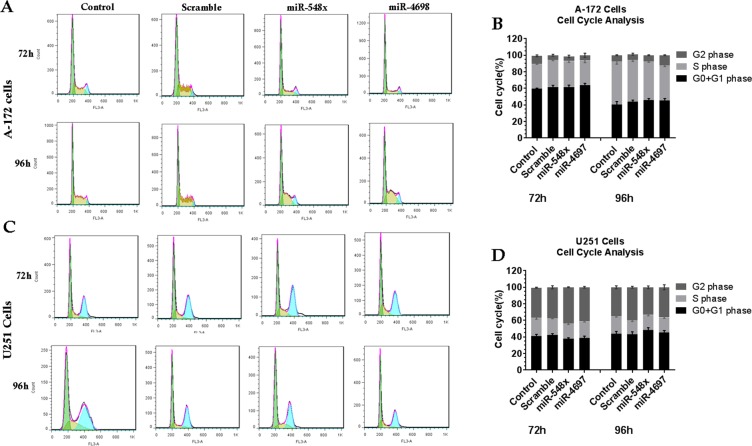
MiR-548x or miR-4698 and the cell cycle arrested of human glioblastoma cell lines. The cell cycle analysis exhibited that miR-548x could not significantly increase cell cycle arrest at G0/G1 phase in U251 (**C,D**) and A-172 (**A,B**) cell lines 72, 96 h post-transduction compared with the respective control. Also, regarding miR-4698 the same result was seen, and no significant change was seen in U251 (**C,D**) and A-172 (**A,B**) cell lines. Data are presented as mean ± SD of results from 3 independent investigations, (^∗^*P* < *0.05*) as compared with the control.

Furthermore, we performed cell cycle analysis to see if these microRNAs are capable of inhibiting the cell cycle. Results showed that overexpression of miR-548x could not significantly increase G0/G1 arrest in the A-172 cells (46.8 ± 1.7% vs. 42.9 ± 1.6%) at 96 h, but was able to increase G0/G1 in U251 cells (49.4 ± 1.4% vs. 43.8 ± 1.6%) as compared to scramble cells (Fig. 8A–D, *P* < *0.05*). Regarding miR-4698 there was no difference between the miR-4698 and the scramble in both cell line A-172 (46.4 ± 1.5% vs. 42.9 ± 1.6%) and U251 (45.9 ± 1.8% vs. 43.8 ± 1.6%) (Fig. 8A–D, *P* > *0.05*).

## Discussion

One of the leading causes of death in humans is cancer. Currently, conventional treatments for cancer (chemo-radiation therapy) provide only relative improvement for patients. Today, there is much research to find an effective treatment for cancer^19^. Gene therapy is the transfer of gene to cells using viral or non-viral vectors. It is predicted that gene therapy in the future will play a vital role in the treatment of cancer^6^.

One of the molecular methods used to treat cancer is RNA interferences. These molecules can attach to the mRNA of target genes and inhibit their translation into proteins. Consequently, the result is a loss of function of the genes. A group of RNA interferences is miRNA, which can simultaneously connect to 3′UTRs of multiple mRNAs and prevent the expression of genes^7,8^. Now, if the candidate miRNA can inhibit a large number of aggressive genes in the PI3K/AKT pathway, it can be used as a gene therapy tool.

GBM features include invasions and resistance to treatment (chemotherapy, radiotherapy). Regardless of the treatment, unfortunately, the average survival rate of patients is less than 15 months, so one of the challenges for oncologists is to find the appropriate treatment for this cancer. Because the cells of the tumor possess resistance to radiation, increased radiation dose due to damage and necrosis in adjacent tissues has little success in treating this type of cancer. Moreover chemotherapy has also been shown to improve survival in only a small subgroup of GBM patients^20,21^. It has been reported that PTEN gene has mutated in about 30% of glioblastoma cases. This gene encodes a protein that has tumor suppressor function and is a negative regulator of the PI3K/AKT pathway by converting PIP3 to PIP2. PTEN gene mutation in glioblastoma includes frameshift, missense, splice-site, and nonsense mutations. The U251 and A-172 cell lines both have PTEN mutant. According to clinical data that were published by Xu *et al*., PTEN nonsense mutation is associated with reduced of Gata3 and p53 protein levels, which this case genomic instability increased in GBM tissues. Since AKT participates in the regulation of various cellular processes, such as cell growth, cell proliferation, cell survival, and angiogenesis, its suppression plays an essential role in the treatment of cancers that the PI3K/AKT pathway is out of control^22–26^.

Increasing evidence has shown that over the past decade many attempts have been made to use microRNAs, because these molecules have clinical potential in cancer therapy. For example, it has been seen that miR-7, miR-34a, miR-223-3p, miR-598, miR-194-5p in glioblastoma cell lines have tumor suppressor function and can reduce growth, proliferation, inhibit cell cycle progression or induce apoptosis^27–30^.

In the present study, we searched for microRNAs that could inhibit the PI3K pathway in glioblastoma cell lines. Although previous studies in TCGA have not reported any expression changes for miR-548x and miR-4698 in glioblastoma^11^, our bioinformatics studies (GSE93850) reveal that the expression of miR-4698 was downregulated (P < 0.05) in the serum of GBM patient compared to the healthy group. Also, another investigation showed (GSE32466, GSE103229, GSE112462) expression of miR-548o-b-a-g-k-d-e downregulated in GBM, but miR-548x and miR-4698 did not show any significant change compared to control. Only we saw (GSE104554) the expression of miR-548x was downregulated (−0.6, P < 0.05) in patients with an under 7-month overall survival rate compared to patients with an upper 15-month overall survival rate. Furthermore, our bioinformatics studies predicted that these two miRNAs could target some PI3K/AKT pathway genes (such as PI3KCA, PI3KCB, AKT, Rheb, mTOR, PDK1). The first step in the treatment of cancer is to reduce viability and cell proliferation. Therefore, in the next step, we studied whether overexpression of miR-548x and miR-4698 could inhibit the viability, proliferation and colony formation of GBM cell lines. The results have shown that these microRNAs could significantly inhibit the proliferation and the viability of GBM cells, which proposed miR-548x and miR-4698 also served as a tumor suppressor miRNA in GBM. However, in the results of the cell cycle and annexin, it was observed that miR-4698 and miR-548x could not confer any significant change compared with control groups.

Our qRT-PCR results showed that overexpression of miR-548x was associated with a reduction in the expression of the PI3KCB, AKT1, mTOR, PDK1, Rheb, and CREB genes. Also, overexpression of miR-4698 was associated with downregulation of the PI3KCA, PI3KCB, AKT1, mTOR, PDK1, Rheb, and CREB genes. Considering that these genes were predicted target genes of miR-548x and miR-4698, Luciferase assay was performed. The results of Luciferase Reporter Assay showed that miR-548x and miR-4698 could reduce the luciferase activity of Rheb, PDK1, and AKT1 genes. Therefore, it can be concluded that the reduction of these genes was due to the direct effect of miR-548x and miR-4698 on their 3′UTR.

In our investigation, we detected that AKT1 was a direct target of miR-548x and miR-4698 in glioblastoma cell lines. Overexpression of miR-548x or miR-4698 was able to significantly downregulate the mRNA and protein levels of AKT1 in A-172 and U251 cell lines. The AKT signaling pathway is overexpressed in many cancers. This pathway plays a critical role in regulating cellular metabolism, growth, proliferation, and development of the tumor. Given that AKT plays an essential role in increasing survival and inhibition of apoptosis, therefore targeting it can play a significant role in the treatment of cancer^31,32^.

In summary, considering the results of this study and the fact that cell proliferation requires protein synthesis, it is predictable that overexpression of miR-548x or miR-4698 can lead to reducing cell proliferation in MTT assay, proliferation assay, and colony formation assays. Nonetheless, according to our findings, the contemporary use of these miRNAs with standard clinical therapies may promote the therapy of glioblastoma tumors. Of course, this needs to be investigated further in the *in vivo*.
